# Antimicrobial use in animal farms in Egypt: rates, patterns, and determinants

**DOI:** 10.1186/s42506-024-00180-w

**Published:** 2025-01-20

**Authors:** Nada ElSayed, Amira Amine, Laila El-Attar, Mohamed E. K. Amin

**Affiliations:** 1 Department of Microbiology, High Institute of Public Health, Alexandria University, Alexandria, Egypt; 2 Faculty of Pharmacy, Alamein International University, Matrouh, Egypt

**Keywords:** Antimicrobial resistance, Antimicrobial use, One Health, Animal farm, Behavior, Theory of reasoned action

## Abstract

**Background:**

While antimicrobial use (AMU) in human healthcare has received significant attention as a key driver of antimicrobial resistance (AMR), less emphasis has been placed on AMU practices and attitudes in animal husbandry. To address this gap, this study examines the patterns and underlying drivers of AMU on animal farms.

**Methods:**

A survey instrument was distributed to farm staff in 150 animal farms across 15 Egyptian governorates. Farms were selected from a list curated by a private platform specialized in Egypt’s poultry and cattle industry. An online search was conducted to identify additional farms not included in the list or when the contact information was unavailable. The instrument covered key items related to AMU including types of antimicrobials used, indications, their perceived benefits, and the feed conversion ratio (FCR). Using the theory of reasoned action (TRA) as a guiding theoretical framework, this study assesses key factors influencing the AMU behavior of farm personnel.

**Results:**

Out of 150 farm personnel invited to fill out the survey forms, 111 (74%) responded and agreed to participate. All surveyed personnel reported using antimicrobials, and almost two-thirds (65%) reported using them for non-therapeutic purposes. Non-therapeutic use of antimicrobials, however, had no impact on FCR across all farm types (poultry, cattle, and mixed). The most used antimicrobials were penicillins (81%), followed by macrolides (78%) and tetracyclines (72%). Half of the studied farms reported using colistin, with more than half of this segment (55%) reporting using it for non-therapeutic purposes. Farm personnel’s AMU behavior was associated with the TRA constructs: perceived benefits of antimicrobials (attitude) and perceived AMU behavior in other farms (subjective norm).

**Conclusions:**

Antimicrobials are unanimously used in animal farms in Egypt, including those classified as last-resort options, such as colistin. Using antimicrobials for disease prevention or growth promotion did not decrease the FCR. Interventions that target the farm personnel’s attitude and the subjective norm should be carried out to change their behavior regarding the use of antimicrobials. Egyptian guidelines for AMU in livestock are urgently needed, along with training to raise awareness of AMR and safer practices. The use of last-resort drugs like colistin should be banned in animal farming, and national surveillance systems should monitor AMU and AMR trends.

**Supplementary Information:**

The online version contains supplementary material available at 10.1186/s42506-024-00180-w.

## Introduction

The global crisis of AMR threatens our ability to treat and prevent serious bacterial infections, leading to hundreds of thousands of deaths each year. If timely action is not taken, mortality rates could rise to millions in the coming decades. A holistic One Health approach is urgently needed to address this rising challenge. This approach acknowledges how closely human, animal, and environmental health are intertwined, especially concerning AMR. The rising rates of antimicrobial-resistant bacteria on livestock farms not only affect animal health but can also spread to humans and contaminate the environment [1, 2].

Numerous studies globally have documented that AMU in farms contributes to the selection of antimicrobial-resistant bacteria. A study across nine European countries linked AMU levels in farm animals to corresponding AMR rates [3]. In the USA and China, animal farms’ water and soil contained significantly higher levels of AMR genes than other nearby environments [4, 5]. High rates of multidrug-resistant (MDR) bacteria have also been reported in various countries, such as 51% MDR *Escherichia coli* (*E. coli*) in Australian pig farms [6] and 100% MDR *E.coli* and *Salmonella enterica* in Egyptian poultry farms [7]. Resistant bacteria in animal farms can transmit to raw food products [8], farm workers [9], and the environment [10].

Rising demand for animal protein has led to increased animal production and a shift towards intensive farming practices [11]. With an expected doubling in pig and poultry production, two-thirds of the future increase in AMU would be in livestock [12]. Farm personnel use antimicrobials both therapeutically, to treat infections, and non-therapeutically, to prevent infections in healthy animals and promote growth [13]. Many routinely administer antimicrobials in mass quantities to the entire herd, even in the absence of sick animals [14]. For growth promotion, they often add sub-therapeutic doses to feed or water to enhance weight gain and improve feed efficiency [15]. Farm personnel usually measure feed efficiency using the feed conversion ratio (FCR), which reflects the kilograms of feed needed for each kilogram of weight gain; a lower FCR indicates better efficiency [16]. Another common practice on animal farms is using antimicrobials instead of essential but logistically challenging biosafety measures, such as vaccination, isolating sick animals, and maintaining proper housing and feed conditions [17]. Various classes of antimicrobials used in livestock are also widely used in human medicine. In 2019, a World Health Organization (WHO) report categorized medically important antimicrobials into “important,” “highly important,” “critically important,” and “highest priority critically important antimicrobials (HPCIAs)” for human medicine [18]. Nevertheless, all these categories are used in animal farming [19]. Notably, colistin (polymyxin E), which is classified as a reserve antimicrobial for managing MDR bacterial infections when other options are unsuitable, continues to be used in livestock to treat infections, prevent disease, and promote growth [20].

The levels of AMU in livestock vary widely across different regions. According to the Food and Agriculture Organization (FAO), only 42 countries have surveillance systems to monitor AMU in food animals [12]. In the USA (2009–2015), sales of medically important antimicrobials for livestock increased by 21%, with consumption nearly three times higher than those of human medicine. Of these, 97% were highly or critically important antimicrobials, and 72% of use in 2015 was for production purposes [21]. In Canada, a 4-year study found that 97% of cattle received medically important antimicrobials, mainly for infection prevention and control [22]. Europe, though stricter in AMU, still shows high variation, with Cyprus, Italy, and Spain using 20 times more antimicrobials than Nordic countries [23]. China, the largest consumer of antimicrobials in animal farming, accounts for 23% of global use, despite regulatory efforts [24, 25]. Similarly, Brazil and other South American countries report high AMU rates [24]. Until 2020, none of the African countries had a national surveillance system for AMU in animal farming, and there is limited evidence that such systems have been implemented since. However, the AMU rate is thought to range between 78 and 100% [26]. A modeling study identified the Nile Delta, Egypt, and Johannesburg, South Africa, as AMU hotspots in Africa [24].

A principal driver of AMU in livestock is the farm personnel’s behavior. Understanding the factors influencing this behavior is essential for implementing effective interventions to reduce inappropriate AMU [27]. The theory of reasoned action (TRA) is a widely used social-cognitive framework that predicts behavior based on two key components: attitude and subjective norm. Attitude refers to how people perceive the benefits of specific behavior, while subjective norm reflects their perception of social pressure, i.e., whether their peers engage in that behavior [28]. In this context, the TRA serves as a theoretical framework to predict the farm personnel’s intention to use antimicrobials nontherapeutically. “Attitude” would be operationalized as the farm personnel’s perceived benefits of non-therapeutic AMU, and “subjective norm,” would be operationalized as their perceived rate of such use in other farms.

Few studies investigated farm personnel’s behavior regarding AMU in livestock farms, and none used a theory-driven approach. A study in Switzerland found that AMU was likely a deliberate action based on perceived benefits rather than a mere habit, with farm personnel’s perception of their AMU compared to others indicating their actual use [29]. In the Netherlands, livestock farmers believed using antimicrobials was more cost-effective than infection control measures [17]. In Cambodia, all farm personnel routinely added antimicrobials to water for both therapeutic and non-therapeutic purposes, believing they were essential for animal health and growth and assuming all other farms did the same [30]. In Egypt, the situation is particularly concerning, with high rates of AMR [7, 31] and limited data on AMU practices. One study reported that all surveyed poultry farms used antimicrobials but did not specify which antimicrobials were used or why [7]. While providing a valuable addition to the literature, previous studies have been limited by small sample sizes, narrow geographic focus, an emphasis on specific farm types (poultry or cattle), or focusing on pets rather than farm animals.

Our study is the second in a broader project addressing AMR and AMU in Egyptian livestock farms. The first paper revealed significantly higher rates of extended-spectrum β-lactamase-producing, fluoroquinolone-resistant, and colistin-resistant *E. coli* in poultry farms compared to cattle farms [31]. Despite the clear link between AMU in livestock and rising AMR, limited research has explored the drivers of AMU on farms. The present study addresses that gap by using the TRA to assess how farm personnel’s attitudes and perceived norms influence AMU decisions. By examining these behavioral factors, the study highlights a critical One Health concern, as inappropriate AMU threatens not only animal health but also human and environmental health. Understanding these drivers is essential for designing targeted interventions to reduce inappropriate AMU and curb the spread of AMR. To our knowledge, this is the first study in Egypt to investigate AMU patterns in poultry and cattle farms, the types of antimicrobials used, their indications, and farm personnel’s AMU behavior. It is also the first to target AMU behavior in livestock, using a theory-driven approach providing deeper insight into this complex issue.

## Methods

### Study design

The study used a cross-sectional descriptive design conducted between October 2018 and May 2019 across 15 governorates in Egypt. Participating farms were purposively selected based on their geographic distribution and aimed to include a variety of farm types (poultry/cattle) to reflect the actual farm distribution in Egypt.

### Sampling frame and sample size

Since there is no official national list of animal farms in Egypt, a list of 1153 farms was compiled instead. It included 1014 farms from a list curated by a private platform, specialized in Egypt’s poultry and cattle industry [32], and 139 farms found online. The search was conducted on Google and social media platforms to identify additional farms not included in the curated list. It utilized keywords in Arabic and English related to the poultry and cattle industry, such as “poultry farms in Egypt,” “cattle farms in Egypt,” and “livestock farms in Egypt.” After excluding 97 farms due to missing or incomplete contact information, the final list consisted of 1056 farms. From this final list, a total of 150 farms were conveniently selected based on their distribution across poultry and cattle, as well as their geographic distribution to represent different regions of the country. For the selected farms, we contacted the key personnel listed, as described in the data collection section. Farms were excluded from the study if they had incomplete contact information or were no longer operational. Additionally, only key individuals directly involved in the management or decision-making processes related to AMU practices on the farms were included; individuals who were unaware of these practices or opted not to participate were excluded.

Based on an official report by the US Department of Agriculture, the rate of AMU in cattle represented approximately 80% [16]. With an expected response rate of 73% (based on a similar study with a comparable methodology) [29], 10% precision, 80% power, and a 95% confidence interval, the minimum required sample size was 147 farms rounded up to 150 farms. The calculation was performed using G*Power for Windows, version 3.1.9.2 (a program written, conceptualized, and designed by Franz, Universitat Kiel, Germany; freely available Windows application software, 2014) [33], employing the *Z*-test for one group difference from constant.

### Instrument design and data collection

A structured instrument, consisting of 5 distinct sections and a total of 20 items, was developed. Items within Sects. 1, 2, 3, and 5 were used for descriptive purposes, and items within Sect. 4 were guided by TRA to understand predictors of factors potentially associated with the non-therapeutic use of antimicrobials in livestock farms in Egypt.

The first section, consisting of five items, gathered information about the respondent’s role on the farm, the individual responsible for antimicrobial prescription and administration, the farm location, the farm type (poultry, cattle, or mixed), and the number of animals/birds (farm scale). Small-scale poultry farms were defined as having up to 5000 birds, while large-scale farms had more than 5000 birds. Similarly, small-scale cattle farms were defined as having up to 50 heads, while large-scale ones had more than 50 heads [34].

The second section, also consisting of five items, focused on biosafety measures, vaccinations, AMU indications, and whether a withdrawal period (WP) is followed on the farm (which is the time that must elapse between giving an animal the last dose of antimicrobial and the slaughtering or food production from that animal to ensure that the food products do not contain antimicrobial residues above the maximum permissible limit [35]). It inquired about the farm’s FCR as the primary indicator used by farm personnel in Egypt to calculate farm productivity.

The third section, consisting of three items, collected information about all types of antimicrobials used on the farm, their indications, and methods of administration. Respondents could choose multiple options from a list of 12 classes/types of antimicrobials commonly used in animal farming and categorized as highly, critically important, HPCIAs or a last resort for human medicine (penicillins, cephalosporins, polypeptides, aminoglycosides, macrolides, lincosamides, tetracyclines, quinolones, sulfonamides, amphenicols, nitrofurantoin, and colistin). This section was designed to assess the actual practices of AMU and non-therapeutic use (behavior).

The fourth section, guided by the TRA, consisted of four items that focused on respondents’ opinions regarding AMU. The first item inquired about respondents’ perceived benefits of non-therapeutic AMU. The second item asked for the respondents’ opinions on the possibility of using alternatives for AMU for non-therapeutic purposes. The third item was presented on a 5-point percentage scale to capture their perceived rate of non-therapeutic AMU in other similar farms (subjective norm). The fourth item was presented on a 4-point Likert scale to reflect respondents’ attitudes towards non-therapeutic AMU (attitude).

Finally, the fifth section, consisting of three items, focused on assessing the feasibility of future interventions. It inquired about their willingness to receive AMU-related training, AMU-related topics of greatest interest, and their preferred method of training. The instrument was developed in Modern Standard Arabic (Fuṣḥa) and is attached, together with an English translation (see supplementary file).

The instrument was distributed to personnel at 150 livestock farms in Egypt, one recipient per farm. Initially, the listed contact person for each farm was contacted through email or phone to explain the study objectives and to get the contact details for the individual responsible for AMU on the farm. Subsequently, that individual was contacted directly to provide further details of the study. Upon obtaining their consent to participate, they were sent the link to the instrument.

### Psychometric properties of instrument

To ensure the instrument’s validity, an expert panel consisting of a social sciences researcher, a senior veterinarian, and a professor of veterinary medicine reviewed the instrument. Based on their feedback, two modifications were made. First, the least socially desirable answers were moved to the top of the list of answer choices to minimize response bias. Second, an additional section was added to inquire about the possibility of future training related to AMR. In addition, a pilot study was carried out on 20 field veterinarians to check the clarity of the instrument items. Two veterinarians reported that they could not view all the answer choices when opening the instrument form on their mobile phones. Thus, the survey form was adjusted accordingly to ensure the accessibility of all the items. Findings also confirmed that participants reported their AMU behavior more accurately than their intentions for AMU in the future. Accordingly, non-therapeutic AMU was used instead of intention for non-therapeutic AMU as the dependent variable in this study. Furthermore, test–retest reliability was examined, where 15 veterinarians filled out the instrument twice with an interval of 15–20 days. Cohen’s kappa was used to measure the level of agreement between the two instances as the instrument primarily consisted of categorical variables. For all the items, the value of Cohen’s kappa ranged from 0.6 to 1, i.e., there was moderate, strong, or almost perfect agreement between the two sets of responses provided by participants across the two incidents. This high level of agreement is essential to ensure that our findings reflect consistent patterns in AMU behavior rather than random variation [36].

### Statistical analysis

We used IBM® SPSS® version 21.0 (IBM, Armonk, NY, USA) to analyze the data. Qualitative data were described by number and percentage. A *p*-value ≤ 0.05 was the cutoff for statistical significance. The chi-square (*χ*^2^) test was used to examine the association between categorical variables, while Fisher’s exact test was applied in cases of small expected frequencies (i.e., when more than 20% of the cells had expected counts of less than 5) and for two-by-two tables. For larger tables, the Monte Carlo exact test was employed, as it allows for computationally intensive calculations due to a greater number of possible outcomes. The Mann–Whitney *U*-test was applied to compare two groups of non-normally distributed quantitative variables. We conducted multiple logistic regression analyses to identify factors potentially associated with non-therapeutic AMU in livestock farms, which represented a clear-cut pattern of injudicious behavior driving AMR. In the multiple logistic regression model, key assumptions were evaluated and confirmed, including the absence of significant residuals, linearity, and multicollinearity. The absence of significant residuals refers to assessing the differences between observed and predicted values. In a well-fitting model, residuals should be small and randomly distributed, indicating the model effectively captures the relationships between variables. Since our independent variables are categorical, typical concerns about outliers are less relevant. However, we examined the casewise listing of residuals and Cook’s distance to ensure no observation had undue influence on the model. While logistic regression does not require a linear relationship between the dependent and independent variables, it assumes a linear relationship between the independent variables and the log odds of the dependent variable. This assumption mainly affects continuous variables and was therefore not a concern for our categorical variables. To assess multicollinearity, we used the chi-square test and calculated Cramér’s V to confirm that the independent variables were not highly correlated, thus ensuring the model’s reliability.

## Results

Out of 150 contacted persons in charge in 150 livestock farms, 111 (74%) responded and agreed to participate, 36 (24%) did not respond, and 3 (2%) responded but refused to participate in the study. On average, it took 11 min to fill in the form. The study included 91% of participants who were solely veterinarians (87%) and both veterinarians and farm owners (3.6%). An additional 5% of the participants were farm owners only, while 4% were farmworkers. Only 60 participants (54%) reported the farm location, where almost half of them (48%) were in the Nile Delta, and the rest were in Giza (18%), Cairo (15%), Alexandria (12%), Minya (3%), Fayoum (2%), and New Valley (2%). Most of the farms (84%) were classified as large-scale farms. Fifty-five (50%) were poultry farms, 43 (39%) were cattle farms, and 13 (12%) were mixed farms with cattle and poultry. No significant difference was detected between cattle, poultry, and mixed farms regarding the participants’ role (*p* = 0.612), the farm location (*p* = 0.264), or the farm scale (*χ*^2^ = 1.27, *p* = 0.529) (Table 1).

**Table 1:** Distribution of participants by role, farm location, farm type, and farm scale among livestock farms in Egypt (2018–2019)

	**Farm type**									*P*
	Cattle		Poultry		Mixed		Total			
	No.	%	No.	%	No.	%	No.		%	
**Role of participant**										0.612¥
Veterinarian	39	91.0	46	84.0	12	92.0	97		87.0	
Owner	2	5.0	3	6.0	1	8.0	6		5.0	
Both vet & owner	2	5.0	2	4.0	0	0.0	4		4.0	
Worker	0	0.0	4	7.0	0	0.0	4		4.0	
Total	43	100	55	100	13	100	111		100	
**Governorate region**										0.264¥
Nile Delta^a^	8	36.0	19	58.0	2	40.0	29	48.0		
Other governorates	14	64.0	14	42.0	3	60.0	31	52.0		
Total	22	100	33	100	5	100	60	100		
**Farm scale**^**b**^										0.529
Large scale	38	88.0	45	82.0	10	77.0	93	84.0		
Small scale	5	12.0	10	18.0	3	23.0	18	16.0		
Total	43	100	55	100	13	100	111	100		

¥Monte-Carlo P

^a^Nile Delta governorates include the following: Beheira, Dakahlia, Damietta, Gharbia, Ismailia, Kafr El-Sheikh, Menofyia, Qalyubia, and Sharqia

^b^For poultry, small scale had up to 5000 birds, while large scale had > 5000 birds, and for cattle, small scale had up to 50 heads, while large scale had > 50 heads

In all farm types, the most common biosafety measure was vaccination (100%), followed by insect and rodent control (80%), isolating sick animals (77%), and regular cleaning (73%). Although all participants reported vaccinating their animals, there was variability in the types of vaccines used. The most common vaccines were against foot-and-mouth disease (79%) and Rift Valley fever (28%) in cattle and avian influenza (36%) and Newcastle (27%) in poultry. The rates of adding antimicrobials to animals’ feed, to animals’ water, and storing small quantities of feed were significantly higher in poultry farms as compared to cattle farms: 24%, 60%, and 65% compared to 5%, 7%, and 28%, respectively (Table 2).

**Table 2:** Distribution of AMU indications and infection control measures among livestock farms in Egypt (2018–2019)

	Farm type								*P*
	Cattle (*n* = 43)		Poultry (*n* = 55)		Mixed (*n* = 13)		Total (*n* = 111)		
	**No.**	**%**	**No.**	**%**	**No.**	**%**	**No.**	**%**	
**AMU indications** ^**a**^									
1. Non-therapeutic uses	18	42.0	48	87.0	6	46.0	72	65.0	< 0.001*
• Increase weight	3	7.0	9	16.0	1	8.0	13	12.0	
• Prophylaxis without the presence of an infected animal(s)	7	16.0	27	49.0	4	31.0	38	34.0	
• Prophylaxis with the presence of infected animal(s)	12	28.0	32	58.0	5	38.0	49	44.0	
2. Therapeutic use (treatment only)	25	58.0	7	13.0	7	54.0	39	35.0	0.096¥
**Biosafety measures**^**a**^									
Vaccinations	43	100	55	100	13	100	111	100	-
Insect/rodent control	31	72.0	48	87.0	10	77.0	89	80.0	0.166
Sick animals’ isolation	36	84.0	38	69.0	12	92.0	86	77.0	0.090
Regular cleaning	27	63.0	44	80.0	10	77.0	81	73.0	0.154
Proper storage conditions	27	63.0	39	71.0	8	62.0	74	67.0	0.641
Regular check on feed (color change, odor change, mold formation)	22	51.0	35	64.0	8	62.0	65	59.0	0.449
Storing feed in small quantities (sufficient for only a few days)	12	28.0	36	65.0	8	62.0	56	50.0	0.001*
Antimicrobials in water	3	7.0	33	60.0	5	38.0	41	37.0	< 0.001*
Antimicrobials in feed	2	5.0	13	24.0	1	8.0	16	14.0	0.022*
**Antimicrobial WP**									
Never	3	7.0	5	9.0	2	15.0	10	9.0	0.725¥
Sometimes	9	21.0	16	29.0	4	31.0	29	26.0	
Always	31	72.0	34	62.0	7	54.0	72	65.0	

*WP *withdrawal period

^*^p ≤ 0.05 (significant). ¥ Monte-Carlo P

^a^The total response percentage exceeds 100% (participants could choose more than one answer)

All participants reported using antimicrobials on their farms. There was a significant difference (*p* < 0.001) in AMU between poultry farms on one hand and cattle and mixed farms on the other hand, where more than 3 quarters of poultry farms (87%) reported using antimicrobials for non-therapeutic purposes, compared to less than half in cattle and mixed farms (42% and 46%). Large-scale farms had a higher rate of non-therapeutic AMU; however, the association was statistically significant in poultry farms only, where 87% of the large-scale poultry farms used antimicrobials for non-therapeutic purposes compared to 56% of the small-scale ones (*p* = 0.015).

**Fig. 1:**
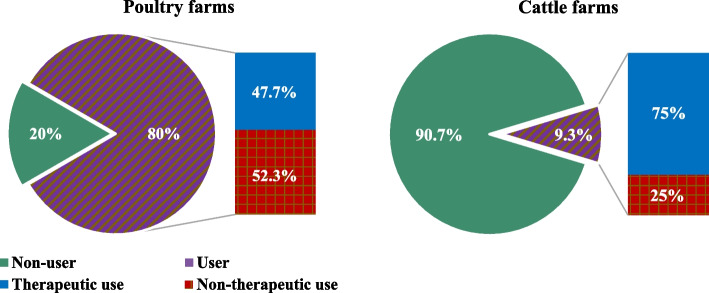
Rates and indications of AMU for various antimicrobial types in livestock farms across Egypt in 2018–2019

Farm personnel reported using penicillins (81%), followed by macrolides (78%) and tetracyclines (72%). Half (50%) of the studied farms reported using colistin, of which 55% reported using it for non-therapeutic purposes (Fig. 1).

**Fig. 2:**
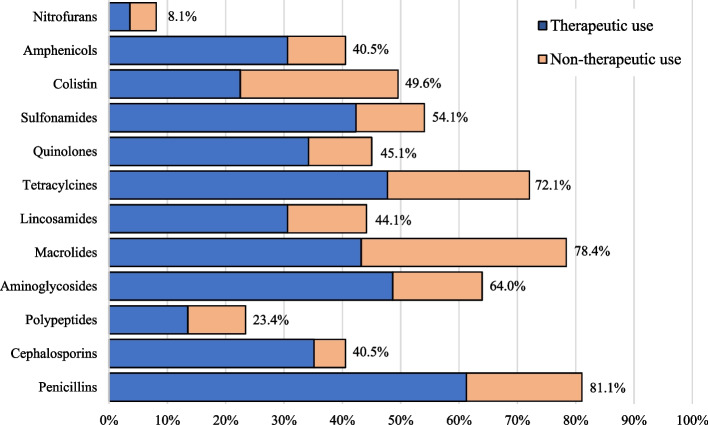
Rates of colistin use in poultry and cattle farms across Egypt in 2018–2019

Poultry farms had a significantly higher rate (*p* < 0.001) of reported total and non-therapeutic colistin use (80% and 52%, respectively) than cattle farms (9% and 25%, respectively) (Fig. 2). Oral administration was the main route of administration for all the mentioned antimicrobials (70–100%), except for cephalosporins, where parenteral administration was the main route (80%). Additionally, adding antimicrobials to the animals’ drinking water was the single most common route for colistin (96%), macrolides (51%), tetracyclines (50%), polypeptides (50%), penicillins (49%), lincosamides (43%), and quinolones (40%).

More than one-third (35%) of the farms, either always (9%) or most of the time (26%), did not leave an antimicrobial WP before slaughtering or using animal products. The percentage was higher in poultry and mixed farms than in cattle farms, but the difference was not statistically significant (Table 2). FCR in poultry farms was mainly below 2 (81%) with a median of 1.6, while in cattle farms it was mostly above 4 (77%) with a median of 5. There was no statistically significant association between the indications for AMU and the FCR in both cattle (*p* = 0.948) and poultry farms (*p* = 0.558). The median FCR in cattle farms was the same with both therapeutic and non-therapeutic AMU. Similarly, the median FCR in poultry farms was 1.8 and 1.6 with therapeutic and non-therapeutic AMU, respectively.

Most respondents (96%) believed that antimicrobials are beneficial for treating sick animals, while less than half (46%) considered them beneficial for prophylactic use. A smaller proportion (15%) thought antimicrobials were helpful for increasing production or profit, and 14% believed they provided protection for farm personnel. When it came to alternatives to non-therapeutic AMU in livestock, over half of the participants (54%) thought it could be partially replaced, and 22% believed it could be completely replaced. Furthermore, nearly one-third of respondents (31%) found non-therapeutic AMU to be not useful at all. Approximately, one-fifth of participants (21%) thought that 80–100% of other livestock farms used antimicrobials for non-therapeutic purposes (Table 3).

**Table 3:** Participants’ perceptions about the benefits of antimicrobials, replacement of antimicrobials for non-therapeutic use, non-therapeutic AMU in other farms, and usefulness of antimicrobials in livestock farms in Egypt (2018–2019)

Participants’ perceptions (*n* = 111)	No.	%
**Perceived benefits of AMU**^**a**^		
Treatment	107	96.0
Prophylaxis	51	46.0
Increase production	17	15.0
Increase profit	17	15.0
Workers’ protection	15	14.0
**Perceived possibility of using alternatives to antimicrobials for growth promotion and disease prophylaxis**		
No	27	24.0
Yes, partially	60	54.0
Yes, completely	24	22.0
**Perceived usefulness of non-therapeutic AMU**		
Very useful	7	6.0
Useful	21	19.0
Slightly useful	49	44.0
Not useful at all	34	31.0
**Perceived rates of non-therapeutic AMU in other similar farms**		
No idea	22	20.0
≤ 20%	27	24.0
21–40%	11	10.0
41–60%	12	11.0
61–80%	16	14.0
81–100%	23	21.0

^a^The total response percentage exceeds 100% (participants could choose more than one answer)

Multiple logistic regression revealed that perceiving non-therapeutic AMU as having some usefulness (slightly useful or useful), compared to non-useful at all, and thinking that more than 60% of the other farms use antimicrobials for non-therapeutic purposes were significantly associated with a higher rate of non-therapeutic use of antimicrobials. The odds of non-therapeutic AMU increased with increased perception of non-therapeutic AMU usefulness. The odds of AMU were 4.75 times and 6.16 times higher when perceived as slightly useful or useful, respectively, compared to perceiving it as not useful at all. Additionally, thinking that 61–80% and 81–100% of the other livestock farms used antimicrobials for non-therapeutic purposes increased the odds of non-therapeutic AMU by 26.90 and 14.79 times, respectively (Table 4).

**Table 4:** Multiple logistic regression of factors potentially associated with non-therapeutic use of antimicrobials in livestock farms in Egypt (2018–2019)

Factors	*P*	OR^a^	95% *CI*	
			**Lower**	**Upper**
**How useful is non-therapeutic AMU for your farm?**				
Not useful at all		1		
Slightly useful	0.003*	4.749	1.712	13.174
Useful	0.011*	6.162	1.510	25.156
Very useful	0.182	3.972	0.524	30.130
**Perceived percentage of other farms, similar to the respondent’s farm, using antimicrobials for non-therapeutic purposes**				
No idea		1		
≤ 20%	0.056	4.32	0.96	19.42
21–40%	0.629	1.54	0.27	8.78
41–60%	0.709	1.41	0.23	8.75
61–80%	0.008*	26.90	2.33	310.80
81–100%	0.006*	14.79	2.17	100.83
Constant	0.010	0.02		

The Hosmer and Lemeshow chi-square test indicated a good model fit (chi-square = 2.78, *p* = 0.905). The classification table showed 76.6% agreement between actual and predicted group membership (cutoff = 0.50). Bootstrap analysis (1000 samples) confirmed the model’s stability

^*^p < 0.05 (significant)

^a^*OR* odds ratio (when > 1 indicates a direct proportion between the assessed factor and non-therapeutic use of antimicrobials, whereas < 1 indicates an inverse proportion between them)

Concerning training related to AMU in livestock, four in five participants (80%) reported their willingness to receive training, preferably as an online course (69%) or a workshop (56%), and to cover the topics of the proper use of antimicrobials in farm animals (60%), prophylaxis against infections in farm animals (31%), and AMR (29%).

## Discussion

AMU in livestock farming, particularly in poultry and cattle, poses a significant challenge due to its contribution to the development of AMR. Understanding the patterns and behaviors associated with AMU is crucial in devising effective interventions to rationalize its use and mitigate the public health threat of AMR.

Key study findings indicate that all respondents reported using antimicrobials on their farms, which aligns with previous findings [7, 25]. Half of them reported the use of colistin, of which 55% reported they used it for disease prevention and growth promotion. Alarmingly, quite often, participants do not leave a WP between using antimicrobials and slaughtering or using animal products. Poultry farms had significantly higher rates of non-therapeutic AMU, total colistin use, and non-therapeutic colistin use than cattle farms. Our results also showed that the farm personnel’s attitudes towards non-therapeutic AMU and their perceived rates of non-therapeutic AMU in similar farms were associated with their actual non-therapeutic AMU.

During this study, several Egyptian websites were found to provide fixed schedules for growing poultry, which included routine antimicrobial administration. Those schedules repeatedly included ciprofloxacin and colistin administration to poultry from day 1. That practice might have had repercussions reflected in the high resistance rates of *E. coli* against these two antimicrobials found in our previous work in Egypt (81% and 44% for ciprofloxacin and colistin, respectively) [31]. It is concerning that many participants did not consistently observe WP before slaughtering or using animal products, a practice that may lead to antimicrobial residues entering the food chain. Similarly, Xu et al. in China found that 26% of poultry farmers did not follow a WP [25]. The lack of a WP results in antimicrobial residues reaching consumers and exposing them to hazardous adverse effects [35].

AMU in poultry farms was more injudicious than in cattle farms. Van Boeckel et al. statistically predicted a global average rate of AMU in poultry three times higher than in cattle [24]. Moreover, Xu et al. and Glasgow et al. found that 78% and 88% of poultry farmers in China and Grenada, respectively, used antimicrobials for non-therapeutic purposes, and the latter reported routine antimicrobial administration to chickens since early life [25, 37]. Poultry is thought to be more prone to *Salmonella* and *E. coli* infections; hence, antimicrobials targeting gram-negative bacteria are heavily used in poultry farms [38]. Additionally, in the studied poultry farms, large-scale farms had a significantly higher rate of non-therapeutic AMU (87%) than small-scale ones (56%). That comes in line with the poor AMU practices reported with intensive animal farming, where it might not be feasible to individualize AMU and where routine mass antimicrobial administration is a common practice [38]. The situation is more challenging in large-scale poultry farms, probably because they have an even larger number of animals than cattle farms [34].

The WHO guidelines for appropriate AMU in food animals recommend a total cessation of using medically important antimicrobials as growth promoters and restricting their therapeutic use to medically diagnosed infections. Moreover, critically important antimicrobials should not be used for prophylaxis, and HPCIAs should be avoided entirely [39]. However, in our study, participants reported that the most commonly used antimicrobials on their farms were penicillins, macrolides, and tetracyclines. Penicillins are classified as highly important for human medicine, tetracyclines are critically important, and macrolides are classified as HPCIAs [18]. In addition, half of the studied farms used colistin, mostly for non-therapeutic purposes. It is concerning that colistin is also one of the HPCIAs and is used as a last resort for treating serious antimicrobial-resistant infections [39]. Furthermore, poultry farms had significantly higher rates of both total and non-therapeutic colistin use than cattle farms. That might be explained by the observed online dissemination of information on social media (Facebook groups, blogs, websites, etc.), recommending colistin several times during the rearing cycle in poultry. Also, colistin is legally sold in Egypt for veterinary use and is available via several pharmaceutical companies, making it accessible and relatively easy to procure [40].

Another important factor is the route of antimicrobial administration. Oral administration was the main route for all antimicrobial classes, except for cephalosporins. Also, adding antimicrobials to the animal’s drinking water was the single most common route for colistin, macrolides, tetracyclines, polypeptides, penicillins, lincosamides, and quinolones. In animal farming, especially in large-scale settings, this sometimes can be the most feasible route. However, it poses a risk of improper dosing due to seasonal variations; for example, during summer, higher temperatures lead to water evaporation, increased antimicrobial concentration, and higher consumption by animals. That is added to the risk of selective pressure on gut microbiota induced by oral antimicrobial administration, thus contributing to the development of AMR [19]. The European guidelines recommend against mass AMU and state that individual drug administration should be applied whenever possible [41].

Although all the participants reported vaccinating their animals, there was vast variability in vaccine types. The Egyptian Central Administration for Veterinary Quarantine often carries out vaccination campaigns, against foot-and-mouth disease, Rift Valley fever, and avian influenza, in cattle and poultry farms and livestock markets. However, these campaigns usually take place in response to epidemics [42]. There is currently no fixed vaccination schedule for farm animals in Egypt.

Among methods used to measure livestock farm productivity, FCR is one of the most common indicators. A lower FCR indicates greater farm productivity [16]. In the participating farms, the FCR values were consistent with the normal ranges reported in Canada: below 2 for poultry and 4.5–7 for cattle [43]. It is noteworthy that no significant difference was found in median FCR between farms that used antimicrobials therapeutically and those that used them nontherapeutically, for both poultry and cattle. That is consistent with a report published in the USA, where there was no significant difference regarding the FCR, the annual production, and mortality rate between poultry farms that used antimicrobials for non-therapeutic purposes versus those that did not. The report suggested a decline in the efficacy of antimicrobials as growth promoters since 2000 [16].

It is essential to understand the drivers behind AMU in livestock [27]. We found a significant association between the participants’ attitudes towards non-therapeutic AMU and the actual use rate in their farms. Also, there was a highly significant association between the participants’ perception of the rate of non-therapeutic AMU in other farms (subjective norm) and the non-therapeutic AMU in their own farms. Non-therapeutic AMU was significantly higher among those who thought that most other livestock farms used antimicrobials for non-therapeutic purposes. A similar association was reported by Visschers et al. and Om and McLaws, who found that antimicrobial was influenced by how the farm personnel perceived the AMU rates in other farms [29, 30].

It is promising that most participants agreed to get training, particularly on proper AMU in animal farming, protecting animals against infections, and AMR. This agreement may indicate their awareness of the problem and a willingness to implement future interventions to rationalize AMU on their farms. It is worth mentioning that Xu et al. found that prior training of livestock farm personnel was associated with better AMU [25]. Future training can be tailored to accommodate different farm personnel through a variety of approaches, such as online courses, social media campaigns, and awareness campaigns at the farms. They can include reaching out to persons managing Facebook pages and blogs with a large audience of farm personnel and incorporating awareness campaigns into vaccination campaigns conducted by the Egyptian Central Administration for Veterinary Quarantine at the farms. Topics to be covered can include essential biosafety measures, the AMR implications, and the farm personnel’s roles in preventing and combating AMR. To promote more effective and sustainable farming practices, interventions should be developed to educate farmers on the benefits of judicious AMU and alternative growth-promoting methods, as well as the repeatedly reported finding that non-therapeutic AMU does not improve feed efficiency as intended. Furthermore, providing incentives such as subsidies or tax breaks to farms that adhere to proper AMU and biosafety measures and conducting follow-ups on their production rates can help ensure their financial sustainability. These strategies can be augmented by sharing success stories from similar farms to encourage the adoption of best farming practices.

Finally, the current study was the first to shed light on the patterns of AMU by applying a theory-driven approach. The study sample exhibited a distribution of farm locations that mirrored the actual farm distribution in Egypt, as reported by Egypt’s Central Agency for Public Mobilization and Statistics (CAPMAS) in 2019. The CAPMAS report highlights a distinct concentration of poultry production within the Nile Delta governorates, while cattle production tends to be centralized in other regions, similar to the distribution observed in this study (58% of poultry farms vs 36% of cattle farms were in the Nile Delta) [44].

### Study limitations

This study had limitations that are worth discussing. One was the absence of an official farm list in Egypt, necessitating a convenience sampling approach that could introduce selection bias. To mitigate this, we selected farms from various governorates across the country and ensured that the chosen farm types (cattle/poultry) matched the distribution reported in the CAPMAS report [44]. Additionally, there was a possibility of underreporting inappropriate AMU due to social desirability bias, where some participants may have tended to choose more socially favorable answers instead of the true ones. To minimize this, the instrument was self-administered by participants, and confidentiality was assured.

Another limitation is the potential underrepresentation of farms without registered veterinarians. According to the FAO, almost 50% of the poultry farms in Egypt are unregistered, and consequently not subject to governmental supervision [34]. In our study, most of the participants (91%) were veterinarians. This might not be the actual case in most farms in Egypt, where farmers often avoid hiring veterinarians to reduce costs and attempt to treat their animals themselves [7]. If having a veterinarian on staff contributes to reducing injudicious AMU, the actual rates of AMU could be even higher than what was reported here, raising further concerns.

The regulatory framework for AMU in livestock in Egypt is evolving slowly. A recent ministerial decree mandates veterinarian supervision for newly registered farms [45], a step in the right direction, which should be complemented by other policies to reduce the injudicious AMU in animal farms. This requires collaborative efforts of the involved institutions, such as the Ministry of Agriculture and Land Reclamation (MALR), the Ministry of Health and Population (MOHP), and international collaborations with organizations such as the WHO and the FAO. In 2018, Egypt developed a National Action Plan (2018–2022) in collaboration with the WHO to combat AMR using a One Health approach. While some of the plan’s goals related to human health have been achieved, there is currently no evidence that any of the objectives for livestock have been met [46].

## Conclusions

The present study aimed to determine the patterns of AMU in animal farming in Egypt and the behavioral drivers behind it. It concluded that antimicrobials are extensively used in livestock farms in Egypt for therapeutic and non-therapeutic purposes, uncovering key beliefs driving this use. An intervention based on the the TRA can be applied to change the behavior of inappropriate AMU on livestock farms.

There is a need for Egyptian guidelines for AMU in livestock that consider both human and veterinary medical needs. Training and awareness campaigns on AMR, the importance of judicious AMU, essential biosafety measures, and alternative strategies for infection prevention and production increase can help change farm personnel’s attitudes towards AMU. The use of last-resort antimicrobials for human medicine, such as colistin, should be banned in livestock. Such a ban should not only be enforced but also be accompanied by other interventions assisting farm workers in substituting antimicrobials with public health-friendly alternatives. A national surveillance system is needed to assess the trends in AMR and AMU in livestock in Egypt. Future qualitative studies should assess how attitudes and subjective norm beliefs of farm workers are formed and how they impact the decisions for non-therapeutic AMU.

## Supplementary Information

Supplementary Material 1. Survey of Antimicrobial Use in Animal Farms (in Modern Standard Arabic and its English translation).

## Data Availability

The datasets used and/or analyzed during the current study are available from the corresponding author upon reasonable request.

## Abbreviations

AMR: Antimicrobial resistance
AMU: Antimicrobial use
FCR: Feed conversion ratio
HPCIAs: Highest priority critically important antimicrobials
TRA: Theory of reasoned action
WP: Withdrawal period
