# Supplementary material for: Antimicrobial use in animal farms in Egypt: rates, patterns, and determinants
Source: J Egypt Public Health Assoc. 2025 Jan 20;100:1. doi: 10.1186/s42506-024-00180-w (PMC11743412; doi:10.1186/s42506-024-00180-w)
Supplement: Supplementary file 1 — Supplementary Material 1. Survey of Antimicrobial Use in Animal Farms (in Modern Standard Arabic and its English translation). [file 42506_2024_180_MOESM1_ESM.docx]

**Survey of Antimicrobial Use in Animal Farms (in Modern Standard Arabic)**

**القسم الأول:**

1. ما هو دورك في المزرعة (يمكنك أن تختار أكثر من إجابة)؟
   ❒ الطبيب البيطري ❒ صاحب المزرعة ❒أحد العاملين بالمزرعة
2. نوع المزرعة: ❒ دواجن ❒ ماشية ❒ مختلط
3. موقع المزرعة (اسم المحافظة): .............
4. عدد الدواجن/ رؤوس الماشية في المزرعة:....................
5. المسؤول عن وصف المضاد الحيوي للحيوانات بالمزرعة هو (يمكنك أن تختار أكثر من إجابة):

❒ الطبيب البيطري ❒ صاحب المزرعة ❒أحد العاملين بالمزرعة

**القسم الثاني:**

1. ما هي الإجراءات المتبعة لديك في المزرعة للوقاية من العدوى (يمكنك أن تختار أكثر من إجابة):

❒ إضافة مضادات حيوية للعلف. ❒ إضافة مضادات حيوية لماء الشرب.

❒ تخزين العلف في ظروف مناسبة (حرارة، رطوبة، تهوئة،...)

❒ تخزين العلف بكميات تكفي بضعة أيام فقط.

❒ الغسيل والتعقيم الدوري للمعالف والمشارب. ❒ التخلص من الفئران/ الحشرات.

❒ متابعة أي تغير في شكل العلف (تغير اللون، تغير الرائحة، تكون عفن،...)

❒ التحصينات الدورية. ❒ عزل الحيوان المصاب.

❒ أخرى (من فضلك اذكرها):..............................................................

1. إن كنت تقوم بإعطاء تحصينات للحيوانات بمزرعتك، من فضلك اذكرها:

............................................. ...............................................

............................................. ...............................................

1. ما هي أسباب صرف المضادات الحيوية للحيوانات بالمزرعة (يمكنك أن تختار أكثر من إجابة):

❒ تضاف للعلف/للماء لزيادة وزن الحيوانات ❒الوقاية من العدوى عند إصابة أحد الحيوانات

❒ الوقاية من العدوى دون وجود حيوان مصاب ❒ علاج الحيوانات المصابة

❒ أخرى (من فضلك اذكرها):..............................................................

1. هل يتم في مزرعتك ترك فترة لسحب المضاد الحيوي قبل الذبح:

❒ لا ❒ نعم، أحيانا ❒ نعم، دائما

1. ما هو متوسط معدل التحويل لديك في المزرعة (Feed conversion ratio)؟

**(كم كيلوغراما من العلف يستهلكها الحيوان/الطائر ليزيد كيلوغراما واحد من وزنه؟)**

.........................................

**القسم الثالث:**

الجدول التالي يذكر العديد من المضادات الحيوية التي تستخدم عادة لحيوانات المزرعة، برجاء إكمال خانات الجدول أمام جميع الأنواع المستخدمة لديك في المزرعة:

| **(13) طريقة الاستخدام** | | | | | **(12) سبب الاستخدام** | | | 1. **المضاد الحيوي** |  |
| --- | --- | --- | --- | --- | --- | --- | --- | --- | --- |
| مضاف لماء الشرب | مضاف للعلف | بالحقن | موضعي | بالفم | التسمين | العلاج | الوقاية |  |  |
| ❒ | ❒ | ❒ | ❒ | ❒ | ❒ | ❒ | ❒ | **البنيسلينات**  (الأمبيسيلن، الأموكسيسيللين،...) | 1 |
| ❒ | ❒ | ❒ | ❒ | ❒ | ❒ | ❒ | ❒ | **السيفالوسبورينات**  (السيفالكسين، سيفتيوفور، سيفتراياكسون،...) | 2 |
| ❒ | ❒ | ❒ | ❒ | ❒ | ❒ | ❒ | ❒ | **البوليبيبتيدات** (الباسيتراسين،...) | 3 |
| ❒ | ❒ | ❒ | ❒ | ❒ | ❒ | ❒ | ❒ | **الأمينوجليكوزيدات**  (ستربتومايسين، نيومايسين، جنتاميسين، كانامايسين،..) | 4 |
| ❒ | ❒ | ❒ | ❒ | ❒ | ❒ | ❒ | ❒ | **الماكروليدات** (إريثرومايسين، تايلوزين،...) | 5 |
| ❒ | ❒ | ❒ | ❒ | ❒ | ❒ | ❒ | ❒ | **لينكوساميدات** (لينكومايسين، كليندامايسن،...) | 6 |
| ❒ | ❒ | ❒ | ❒ | ❒ | ❒ | ❒ | ❒ | **تيتراسيكلينات** (أوكسي سيكلين، كلورتيتراسيكلين، دوكسي سيكلين،..) | 7 |
| ❒ | ❒ | ❒ | ❒ | ❒ | ❒ | ❒ | ❒ | **الكينولونات** (حمض النالدكسين، سيبروفلوكساسين، نورفلوكساسين،...) | 8 |
| ❒ | ❒ | ❒ | ❒ | ❒ | ❒ | ❒ | ❒ | **السلفوناميدات** (سلفاديازين، سلفا و ترايمثوبريم،...) | 9 |
| ❒ | ❒ | ❒ | ❒ | ❒ | ❒ | ❒ | ❒ | **أمفينيكولات**  (كلورأمفينيكول، فلورفينيكول، ثيامفينيكول،...) | 11 |
| ❒ | ❒ | ❒ | ❒ | ❒ | ❒ | ❒ | ❒ | **الكوليستين** | 10 |
| ❒ | ❒ | ❒ | ❒ | ❒ | ❒ | ❒ | ❒ | **نيتروفيوران** | 12 |
| ❒ | ❒ | ❒ | ❒ | ❒ | ❒ | ❒ | ❒ | **أخرى**:......................... | 13 |

**القسم الرابع:**

1. في رأيك، ما هي فوائد استخدام المضادات الحيوية في حيوانات المزارع (يمكنك أن تختار أكثر من إجابة):

❒ زيادة الإنتاج (زيادة كمية اللحم). ❒ زيادة العائد المادي.

❒ حماية الحيوانات السليمة من الأمراض. ❒ حماية العاملين بالمزرعة من العدوى.

❒ علاج الحيوانات المصابة. ❒ أخرى (اذكرها): ........................................................

1. في اعتقادك، ما مدى الفائدة العائدة على مزرعتك من استخدام المضادات الحيوية بغرض التسمين أو الوقاية من الأمراض؟

| غير مفيد على الإطلاق  Ο | مفيد بعض الشيء  Ο | مفيد  Ο | مفيد جداً  Ο |
| --- | --- | --- | --- |

1. في اعتقادك، ما هي نسبة المزارع الأخرى -المماثلة لمزرعتك- التي تستخدم المضادات الحيوية بغرض التسمين أو الوقاية من الأمراض؟

| 20% أو أقل من المزارع  Ο | 21- 40% من المزارع  Ο | 41- 60% من المزارع  Ο | 61- 80% من المزارع  Ο | 81- 100% من المزارع  Ο |
| --- | --- | --- | --- | --- |

1. في رأيك، هل يمكن استخدام بدائل للمضاد الحيوي في التسمين والوقاية من الأمراض؟

❒ لا ❒ نعم، يمكن بشكل جزئي. ❒ نعم، يمكن بشكل كامل.

**القسم الخامس:**

1. هل ترغب في الحصول علي تدريب عن استخدام المضادات الحيوية في المزارع؟

| Ο نعم | Ο لا |
| --- | --- |

1. ما هي وسيلة التدريب التي تفضلها ؟) يمكن اختيار أكثر من إجابة لهذا السؤال)

| Ο | لا أرغب في الحصول على تدريب |
| --- | --- |
| Ο | حضور ورشة عمل |
| Ο | دورة تدريبية على الانترنت |
| Ο | مطبوعات توزع على العيادات/الوحدات البيطرية |
| Ο | وسيلة أخري (برجاء ذكرها( :........................ |

1. إذا كانت الإجابة بنعم، ما هى الموضوعات التى ترغب في معرفتها أومناقشتها؟

- ......................................................................................................................

---------------------

**Survey of Antimicrobial Use in Animal Farms (English Translation)**

**Section 1:**

1. What is your role on the farm (you can choose more than one answer)?
   ❒ Veterinarian ❒ Farm owner ❒ Farmworker
2. Farm type: ❒ Poultry ❒ Cattle ❒ Mixed
3. Farm location (name of governorate): ………………
4. No. of poultry/cattle heads:....................
5. The person responsible for prescribing antimicrobials on the farm is (you can choose more than one answer):

❒ Veterinarian ❒ Farm owner ❒ Farmworker

**Section 2:**

1. What biosafety measures do you have on the farm? (you can choose more than one answer):

❒ Adding antimicrobials to water ❒ Adding antimicrobials to feed

❒ Proper storage conditions (temperature, humidity, aeration …)

❒ Storing feed in little quantities (sufficient for only a few days)

❒ Regular cleaning (feeders, drinkers …) ❒ Insect/rodent control

❒ Sick animal(s) isolation

❒ Regular check on feed (color, odor, mold growth …)

❒ Vaccinations ❒ Other (please mention): ………………

1. If you administer vaccinations to animals on your farm, please mention them:

............................................. ...............................................

............................................. ...............................................

1. What are the reasons for giving antimicrobials to the animals on the farm? (you can choose more than one answer):

❒ To increase animals’ weight ❒ For prophylaxis with the presence of an infected animal(s)

❒ For prophylaxis without the presence of infected animal(s) ❒ To treat an infected animal(s)

❒ Other (please mention): ………………

1. In your farms, do you leave an antimicrobial-withdrawal period before slaughtering or using animal products?

❒ No ❒ Yes, sometimes ❒ Yes, always

1. What is the average feed conversion ratio on your farm?

**(Number of kilograms of feed consumed by the animal to gain one kilogram of weight)**

.........................................

**Section 3:**

The following table lists antimicrobials that are usually used for farm animals, please check the boxes in front of **all the types used in your farm**:

| **(11) Antimicrobial** | **(12) Indication** | | | **(13) Mode of administration** | | | | | |
| --- | --- | --- | --- | --- | --- | --- | --- | --- | --- |
|  | Prophylaxis | Treatment | Growth promotion | Oral | Topical | Parenteral | Added to feed | Added to drinking water |  |
| 1. **Penicillins** | ❒ | ❒ | ❒ | ❒ | ❒ | ❒ | ❒ | ❒ |  |
| 1. **Cephalosporins** | ❒ | ❒ | ❒ | ❒ | ❒ | ❒ | ❒ | ❒ |  |
| 1. **Polypeptides** | ❒ | ❒ | ❒ | ❒ | ❒ | ❒ | ❒ | ❒ |  |
| 1. **Aminoglycosides** | ❒ | ❒ | ❒ | ❒ | ❒ | ❒ | ❒ | ❒ |  |
| 1. **Macrolides** | ❒ | ❒ | ❒ | ❒ | ❒ | ❒ | ❒ | ❒ |  |
| 1. **Lincosamides** | ❒ | ❒ | ❒ | ❒ | ❒ | ❒ | ❒ | ❒ |  |
| 1. **Tetracyclines** | ❒ | ❒ | ❒ | ❒ | ❒ | ❒ | ❒ | ❒ |  |
| 1. **Quinolones** | ❒ | ❒ | ❒ | ❒ | ❒ | ❒ | ❒ | ❒ |  |
| 1. **Sulfonamides** | ❒ | ❒ | ❒ | ❒ | ❒ | ❒ | ❒ | ❒ |  |
| 1. **Amphenicols** | ❒ | ❒ | ❒ | ❒ | ❒ | ❒ | ❒ | ❒ |  |
| 1. **Colistin** | ❒ | ❒ | ❒ | ❒ | ❒ | ❒ | ❒ | ❒ |  |
| 1. **Nitrofuran** | ❒ | ❒ | ❒ | ❒ | ❒ | ❒ | ❒ | ❒ |  |
| 1. **Others (………..)** | ❒ | ❒ | ❒ | ❒ | ❒ | ❒ | ❒ | ❒ |  |

**Section 4:**

1. In your opinion, what are the benefits of using antimicrobials in farm animals (you can choose more than one answer):

❒ Increased production (increased amount of meat) ❒ Increase profits

❒ Protect healthy animals from diseases ❒ Protecting farm workers from infection

❒ Treating infected animals ❒ Others (please mention):................................

1. In your opinion, can alternatives to antimicrobials be used for weight increase and disease prophylaxis?

❒ No ❒ Yes, partially ❒ Yes, completely

1. In your opinion, what percentage of other farms – that are similar to yours - that use antimicrobials for growth promotion or disease prophylaxis?

| 20% or less of farms  Ο | 21- 40% of farms  Ο | 41- 60% of farms  Ο | 61- 80% of farms  Ο | 81- 100% of farms  Ο |
| --- | --- | --- | --- | --- |

1. In your opinion, how useful to your farms is the use of antimicrobials for growth promotion or disease prophylaxis?

| Not useful at all  Ο | A little useful  Ο | Useful  Ο | Very useful  Ο |
| --- | --- | --- | --- |

**Section 5:**

1. Would you like to get training on the use of antimicrobials on livestock farms?

| Ο No | Ο Yes |
| --- | --- |

1. What is your preferred method of training? (You can choose more than one answer to this question)

| Ο | I do not want to get training |
| --- | --- |
| Ο | Attend a workshop |
| Ο | Online training course |
| Ο | Flyers/posters distributed to veterinary clinics/units |
| Ο | Other means (please specify): ………………. |

1. If you answered yes, what topics would you like to know about or discuss?

………………………………………………………………………….

----------------------
